# Preparing a Personalized Meal by Using Soy, Cricket, and Egg Albumin Protein Based on 3D Printing

**DOI:** 10.3390/foods11152244

**Published:** 2022-07-27

**Authors:** Farnaz Mirazimi, Jordi Saldo, Francesc Sepulcre, Alvar Gràcia, Montserrat Pujolà

**Affiliations:** 1 Departament d’Enginyeria Agroalimentària i Biotecnologia Castelldefels, Campus del Baix Llobregat, Universitat Politècnica de Catalunya, 08860 Castelldefels, Spain; farnaz.sadat.mirazimi@upc.edu (F.M.); francesc.sepulcre@upc.edu (F.S.); montserrat.pujola@upc.edu (M.P.); 2 Centre d’Innovació, Recerca i Transferència en Tecnologia dels Aliments (CIRTTA), XaRTA, CERTA-UAB TECNIO, Animal and Food Science Department, Facultat de Veterinària, Universitat Autònoma de Barcelona, Cerdanyola del Vallès, 08193 Barcelona, Spain; alvar.gracia@gmail.com; 3 Instituto Politécnico Nacional (IPN), Centro de Desarrollo de Productos Bióticos (CEPROBI), Yautepec 62739, Mexico; 4 Natural Machines Iberia, S.L., 08013 Barcelona, Spain

**Keywords:** protein, fortified food, nutrition, texture, rheology, 3D food printing

## Abstract

Recently, personalized meals and customized food design by means of 3D printing technology have been considered over traditional food manufacturing methods. This study examined the effects of different proteins (soy, cricket, and egg albumin protein) in two concentrations (3% and 5%) on rheological, textural, and 3D printing characteristics. The textural and microstructural properties of different formulations were evaluated and compared. The addition of soy and cricket protein induced an increase in yield stress (τ₀), storage modulus (G′), and loss modulus (G″) while egg albumin protein decreased these parameters. The textural analysis (back extrusion and force of extrusion) demonstrated the relationship between increasing the amount of protein in the formula with an improvement in consistency and index of viscosity. These values showed a straight correlation with the printability of fortified formulas. 3D printing of the different formulas revealed that soy and cricket proteins allow the targeting of complex geometry with multilayers.

## 1. Introduction

3D food printing is an example of digitalization in the food sector, which is a popular means of developing the capability of the supply and manufacturing chain [1]. 3D food printing is able to formulate personalized nutrition control and customized food design, and is a potential technology to reconfigure a customized food and prototyping tool to facilitate new food product development [2]. Using 3D printing to personalize food nutrition is a field of high importance in research. Food customization is an advantage of 3DFP (3D Food Printing) technology for providing for an individual’s health-nutritional needs, including for athletes, pregnant women, older adults, and people with dysphagia. Recently, nutrition control, personalized medicine, and some therapeutic approaches have changed. The technological developments associated with food engineering, food processing, and consumption patterns are evolving. Moreover, 3DFP applications are growing to fulfill individual needs and for personalized nutrition [3].

For adults, dietary guidelines suggest an acceptable macronutrient distribution range of 45–65% from carbohydrates, 20–35% from fat, and 10–35% from protein, with a recommended dietary allowance of 46 and 56 g/d or 0.8 g/kg body weight of protein [4].

Soenen et al. [5] demonstrated that a high-protein diet can help with body-weight loss and weight maintenance. This also resulted in a larger reduction of body weight and fat mass, and thereafter, since they promote a sustained level of satiety, sustained energy expenditure, sparing of fat-free mass, and increased fat oxidation [6,7].

Soy protein is a very well-known protein rich in essential and non-essential amino acids and has been extensively studied to demonstrate that it can improve physicochemical properties [8]. Soy protein consumption has been associated with many beneficial health effects [9]. Some studies show that soy protein can reduce the circulating levels of total and LDL-cholesterol, modify the taxonomy of the gut microbiota, and improve insulin sensitivity [10,11]. The addition of soy protein increased mechanical strength and the viscosity of food products and can help the self-supporting ability of 3D-printed food [12].

Edible insects can act as an enriching additive when added to food [13]. They are very rich in protein, essential amino acids, vitamins, and minerals, and have recently been attracting the attention of the food industry and researchers for their use in different formulations with different characteristics [14,15]. Nowadays many studies show that crickets (*Acheta domesticus*) have high nutritional value and consumption is safe and is not associated with side effects. This increasingly improves functionality in food formulations and, as an alternative protein, makes them suitable for many food products [16].

Egg white contains many functionally important proteins, such as ovalbumin, ovo-transferrin, ovomucoid, ovomucin, and lysozyme [17]. Ovalbumin has been used extensively in food technology because of its foaming, emulsification, and binding adhesion [18]. In addition, ovalbumin has been found to have antioxidant (protection of linoleic acid and docosahexaenoic acid), antimutagenic and anticarcinogenic immunomodulatory properties [19].

This work focuses on expanding the shape stability, printability, and nutritional profile of formulated purees that can provide new opportunities for leverage of 3D food printing for consumer needs and health. Protein-rich formula is an encouraging food material in the 3D printing sector, although there is still limited accessible information on food proteins for 3D printing. In the present work, protein-rich formulations have been prepared, and different geometries produced by 3D food printing have been studied. The main aims of this study were to (1) investigate the rheological properties and 3D printability of different protein mixtures, (2) evaluate the texture characteristics of new methods, (3) understand the composition of different proteins regarding printability, and (4) persuade people to consume a personalized diet.

## 2. Materials and Methods

### 2.1. Materials

Potato puree powder (Maggi) was purchased in a local supermarket with the nutritional values presented in Table 1.

Soy Protein acid hydrolysate (SPAH) was procured from Sigma-Aldrich (Nitrogen analysis ≥ 12% total = 75% protein, calculated applying a factor of 6.25 g protein/g N). Cricket protein powder 70% protein (*Acheta domesticus*) was obtained from Origen farms, Albacete, Spain. Egg albumin 75% protein was obtained from Avantor^®^. According to the regulation (EC) N.1924/2006, all the formulations were high in protein [20] (Table 2).

### 2.2. Sample Preparation

The same base was applied for all the formulations: the different proteins (soy (SPAH), cricket, and egg albumin) were dissolved or dispersed in 100 mL of distilled water at 2 different levels (3 and 5%) with the addition of 17 g potato powder. The formulations were prepared by mixing 100 mL of distilled water and protein (albumin or cricket) and keeping the mixture in an oven at 40 °C for 30 min to denature the protein. For soy protein (SPAH), the conditions to denature the protein were 90 °C for 30 min. All the samples were kept in an oven with a lid to avoid evaporation. Finally, the dehydrated potato powder was added. Formulations were coded with a letter indicating the protein source (S, C, or A for soy, cricket, or egg albumin, respectively) and a number expressing the amount of protein added (3 or 5%). CS is the code for the control sample, which shares the same formulation but with no protein added, and the preparation condition was in an oven at 40 °C for 30 min.

Samples were kept overnight (22–24 h) in a fridge set at 2 °C and all samples were placed in an incubator at 20 °C for two hours before starting the rheological measurements.

The whole experiment was repeated 3 times.

### 2.3. Rheological Measurements

#### 2.3.1. Frequency Sweep Test

The rheological measurements were performed with a rheometer (Rheostress RS1, version 127, Barcelona, Spain) and computer software (HAAKE RheoWin 3 Job and Data Manager Software, Thermo Fisher Scientific, Waltham, MA, USA). Samples were analyzed using a plate-plate geometry with a 1-mm gap between the 35-mm serrated plate (PP60 sensor) and the serrated base for their flow properties. The upper plate was placed into the measuring position, and the surplus sample was trimmed. Measuring rheological properties started after leaving samples to rest for 2 min. Two types of rheological tests were run: a rotational test and a dynamic oscillatory test. The temperature of the rheological tests was unvarying at 20.0 ± 0.1 °C for both measurements. The linear viscoelastic region was identified by a strain sweep test at a shear rate of 0.0025 s^−1^ before attempting the dynamic rheological measurement [21]. Oscillatory tests were performed to determine the strength and stability of the different formulations and explain the behavior of material from 0.1 to 10 Hz to understand how viscous or elastic properties dominated the behavior of the prepared formulations. Rheological properties can be evaluated through the storage modulus (G′), loss modulus (G″), and loss tangent (tan δ) which describes the ratio of the loss modulus to storage modulus (G″/G′). Samples were measured before the 3D printing test.

#### 2.3.2. Rotational Rheological Measurements, Viscosity, and Yield Stress

Shear rate was raised during the first 30 s logarithmically from 0.1 to 10 s^−1^, after being preserved at 10 s^−1^ for 30 s, and finally was cut down logarithmically again to 0.1 s^−1^ over 30 s. For each sample, the shear stress (τ), viscosity (η), and yield stress were calculated following standard procedures [22].

### 2.4. Texture Properties Characterization

#### 2.4.1. TPA

3D printed hexagon shape made of 8 layers using a Foodini^®^ 3D Food Printer machine (Natural Machines Iberia, S.L., Barcelona, Spain) was analyzed by TPA test to determine the textural properties of the printed formulations. Texture profile analyses of printed samples were performed at 20 °C using a Texture Analyser (TA. XT Plus, Stable Micro Systems, Godalming GU7 1YL, UK) with a compression plate (P/100). Double-cycle compression tests were performed at a test speed of 2 mm/s, a post-test speed of 2 mm/s, and compression strain was set at 35%, and a with a time between compressions of 5 s. Each formulation was measured three times per sample. Hardness, chewiness, cohesiveness, adhesiveness, chewiness, and resilience were recorded in one replicate per sample.

#### 2.4.2. Backward-Extrusion Test

The TA. XT Plus, Stable Micro Systems, UK, was used to perform a compression analysis with a 3.5 cm diameter plate probe. The purees were placed in a cylindrical container 5 cm in diameter and 6.5 cm high and filled to 75% of the volume of the container.

For comparison, the method was adjusted to mimic the speed of the printing conditions of the Foodini^®^ (speed of 2 mm/s for a distance of 30 mm).

#### 2.4.3. Force of Extrusion

**Figure 1:**
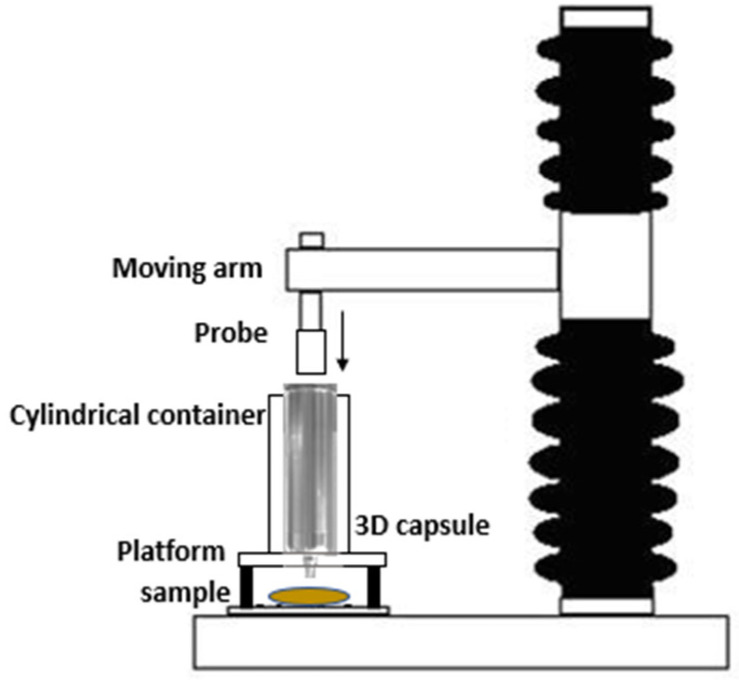
Force of extrusion method.

To determine the firmness (N) and consistency (N·s) of the extrusion process with the Foodini^®^, the 304 stainless steel capsules of the printer (© 2021 Natural Machines), with a capacity of 100 mL and 4 mm mouthpiece have been used with a TA. XT (Stable Micro Systems, UK) was equipped with a 2.5 cm diameter cylindrical probe. The probe applied force to the plunger of the capsule (38 mm in diameter), pushing the puree out through the mouth placed at the bottom of the capsule. The maximum force needed to extrude the product was obtained by the analysis of the results of a test performed at a speed of 2 mm/s and for a distance of 30 mm (Figure 1).

### 2.5. Conditions of 3D Food Printing

For printing, Foodini^®^, an extrusion-based commercial system, was used. The formulations were produced using a nozzle size of 1.5 mm and a hexagon prism of 8 layers was created for printing and analyzing the samples. Before printing the samples all the formulations were passed through a sieve as a means to eliminate or reduce the presence of lumps that may disturb printing, with a nozzle of a small diameter (1.5 mm). Other more complex structures were printed, and if the printed sample’s structures were able to keep their shape for at least 15 min, they were considered printable. The first ingredient holds at 4.2 mm, with the first layer nozzle height at 1.4 mm, and the print speed was set at 3500 mm/min.

### 2.6. Statistical Analysis

Statistical analyses of the data were determined on Minitab 18 (Minitab link., Coventry, UK), using analysis of variance, General Linear Model, and Tukey’s comparison test rheology, and textural characteristics were tested for significant differences (*p*  <  0.05).

## 3. Results

### 3.1. Rheological Properties

#### 3.1.1. Viscosity and Yield Stress

For the formulation to have good printability, the rheological properties of extrusion-based products are critical [23]. For selecting the best performer different mathematical models (e.g., Power Law, Herschel-Bulkley, Casson, and Bingham models) were tested. Bingham, with a high coefficient of determination (R > 0.98), was selected as the best model to fit the flow characteristics of the samples. The Bingham model is explained by the equation τ = τ₀ + ηᵨ ɣ̇, where τ (Pa) is the shear stress, τ₀ (Pa) is the yield stress, ηᵨ (Pa·s) is the viscosity and ɣ̇ (s − 1) is the shear rate.

The addition of SPAH and cricket protein showed a positive correlation with the yield stress. These proteins could increase the mechanical strength in line with other studies [12,24]. The yield stress shows the mechanical strength of formulations. Yield stress is the ability of the formulations to keep their shape under gravity and point to the feasibility of extrusion. They also supported successive stacked layers after deposition. The larger the yield stress, the better the self-supporting, and the easier the smooth extrusion properties [1,25]. The formulation S5 and C5 presented the highest yield stress (535 Pa) and viscosity (451 Pa) among the tested formulations (Table 3) and the lower yield stress and viscosity belongs to the samples with egg albumin protein that could not self-support multilayer shapes, so that the product collapsed after printing.

Table 3 shows that SPAH and cricket had a significant (*p*  <  0.05) effect on raising the viscosity. Increasing the concentration of SPAH had a significant effect on raising viscosity but increasing the amount of cricket and egg albumin from 3% to 5% did not change the viscosity.

The high yield point and viscosity produced through the bonds between protein-starch and protein–protein internal microstructure, contribute to the elasticity of the network of the potato starch puree and consequently generate a starch internal microstructure that is more insistent to deformation. The formulations C5 (917 Pa), C3 (742 Pa), and S3 (636 Pa) showed high yield stress and viscosity (397 Pa), (302 Pa), and (254 Pa), respectively compared to the control sample, which had good results for a multilayer shape.

#### 3.1.2. Viscoelastic Behavior

To investigate the elastic modulus, a frequency sweep test was used. Storage modulus (G′) and loss modulus (G″) of formulations were frequency-dependent revealing a gel-like structure with G′ higher than G″ (Table 4). Storage modulus (G′) indicates the elastic solid-like behavior and the mechanical strength. These values provide information about the mechanical properties that can be connected to the self-supporting capacity of 3D printing [26]. G′ of formulations with SPAH and cricket protein were higher, indicating that they can possess a better load-bearing capacity without deformation.

Food formulations with strong mechanical strength compared to control samples (S5 (995 Pa), C5 (827 Pa), C3 (816 Pa), and S5 (717 Pa)) determine excellent self-supporting performance after deposition and could withstand the complex printed shape.

A high tan δ means more viscous behavior, a low tan δ means more elastic behavior and a tan δ smaller than 1 signifies predominantly elastic behavior. All the formulations were significantly lower than 1 and show viscoelastic behavior.

### 3.2. Texture

#### 3.2.1. TPA

TPA is an important test for textural characterization and is explained by simulating human mastication behavior. It is accomplished as a two-bite compression test as it provides a link between mechanical properties and textural attributes during oral processing [27]. Table 5 shows results of the texture profile analysis (TPA) that was carried out for the printed food samples.

The hardness, cohesiveness, springiness, gumminess, chewiness, and resilience of different proteins had no significant (*p* < 0.05) differences, even when increasing the amount of protein concentration. The only parameter that changed in this test was adhesiveness where the lowest measurement belongs to the A5 (−3.80 N·s) and A3 (−3.73 N·s), respectively, and the highest measurements were for C5 (−5.75 N·s), S5 (−5.70 N·s) and S3 (−5.55 N·s). The other formulations (C3, and CS) showed the same behavior and had no significant differences (*p* < 0.05). The formulations prepared with egg albumin had a relatively low yield point and storage modulus which, considering high adhesiveness, can be translated into the lower energy needed to make a swallowable bolus and weak gel structure [28].

#### 3.2.2. Back Extrusion

Back extrusion is a technique that is used to determine the flow properties of Newtonian and non-Newtonian fluids in the industrial environment for several purposes, including quality control. Some studies show some correlation between the back-extrusion technique and rheological tests [29]. Comparing the measurement of force applied by the annular space with the rheological properties of the samples, it can be observed that the force reflected the rheological properties of the samples. The consistency of samples with SPAH 5% and cricket 5% was higher than the rest of the formulations due to higher resistance to flow and therefore higher shear viscosity.

The index of viscosity is the extrusion energy or work of adhesion when it becomes higher; more resistance is required when pulling out the sample and it has been demonstrated that the index of viscosity is related to the consistency of the purees [30]. The formulations with SPAH had the highest index of viscosity followed by the samples with cricket protein, which had a higher index of viscosity than the control sample. The formulations with albumin showed a low index of viscosity due to the less viscosity and yield stress referring to the rheological test (Table 3).

**Figure 2:**
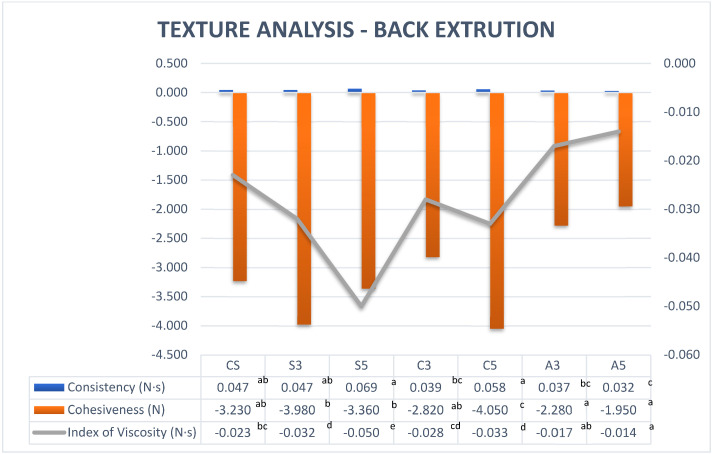
Back extrusion measurements for different proteins at different concentrations. Note: CS (Control Sample), S3 (3% Soy), S5 (5% Soy), C3 (3% Cricket), C5 (5% Cricket), A3 (3% Albumin), and A5 (5% Albumin). Different superscript letters in the same row indicate a significant difference (*p* < 0.05).

Cohesiveness means ‘work of cohesion’, and the more negative the value, the more ‘cohesive’ is the sample. The highest cohesiveness among the tested formulations is for sample C5 and significantly (*p* < 0.05) changed when increasing cricket protein from 3% to 5%. Samples with SPAH (S5 and S3) showed a different behaviour and did not change when increasing the amount of protein from 3% to 5% (Figure 2). The albumin had weak consistency and cohesiveness, which means less resistance to flow.

#### 3.2.3. Force of Extrusion

**Figure 3:**
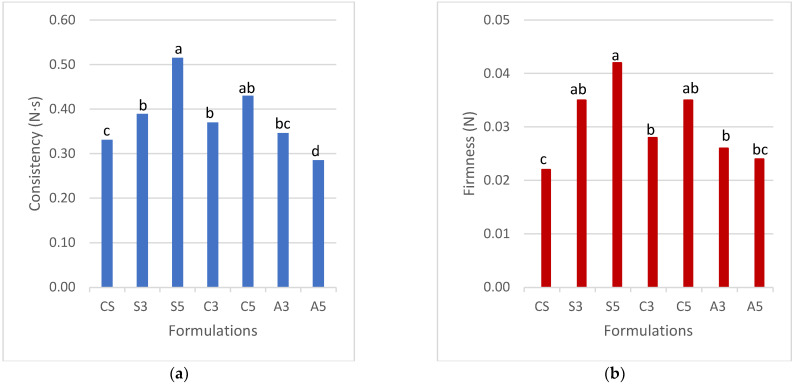
Force of extrusion measurements for different proteins at different concentrations. a) Consistency b) Firmness Note: CS (Control Sample), S3 (3% Soy), S5 (5% Soy), C3 (3% Cricket), C5 (5% Cricket), A3 (3% Albumin), and A5 (5% Albumin). Different superscript letters indicate a significant difference (*p* < 0.05).

According to the force of extrusion method (Figure 3), the formulation S5 had the highest consistency and firmness (*p* < 0.05). When increasing the amount of SPAH from 3 to 5%, these values increased (*p* < 0.05). The formulations with cricket protein (C3 and C5) also increased the consistency (0.37 and 0.43 N·s) and firmness (0.028 and 0.035 N), respectively, compared to the control sample (CS). However, the samples with SPAH and cricket all showed good printability for multilayer shapes. This good performance can be explained by the strong connections between starch granules and protein, with ability to build a 3D network in the case of using soy and cricket protein. Huang et al. [31] showed that the mixtures of the protein and polysaccharides were able to increase the texture parameters that may be described similarly in the case of the mixture of proteins and starch in this work. The formulations with albumin A3 and A5 had poor consistency and firmness pointing to a difficulty in building a strong enough network with the potato starch, as opposed to the case of soy or cricket.

### 3.3. 3D Printing Test and Correlations

**Figure 4:**
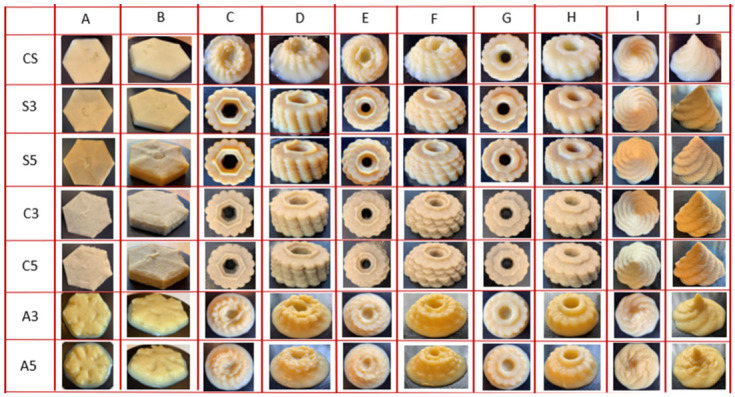
Pictures of 3D printing formulations with different proteins. Note: CS (Control Sample), S3 (3% Soy), S5 (5% Soy), C3 (3% Cricket), C5 (5% Cricket), A3 (3% Albumin), and A5 (5% Albumin). (**A**) 8 layers hexagon shape top (**B**) 8 layers hexagon shape side (**C**) 13 layers flower shape top (**D**) 13 layers flower shape side (**E**) 11 layers flower shape top (**F**) 11 layers flower shape side (**G**) 9 layers flower shape top (**H**) 9 layers flower shape side (**I**) 28 layers mountain shape top (**J**) 28 layers mountain shape side.

As observed in the printing experiments, all the formulations could be easily extruded from the nozzle at 25 °C. To compare the printability and stability of the formulations with different proteins, different designs were applied. Figure 4 shows the result of 3D printing. The formulations with SPAH and cricket protein were able to support the extruded materials and stable self-supporting structures for all the shapes that have been designed, even for the complex examples with 13- and 11-layer flower shapes. The hexagonal shape for all food formulations as well as the control sample was able to maintain the structure for more than 15 min, except for formulations with egg albumin that showed very poor resolution due to the low rheological and textural properties. The control formula, as well as those including SPAH or cricket, were able to form stable self-supporting structures when printing complex flower or mountain shapes (Figure 4G–J) of 9 printed layers. Conversely, the formulations fortified with albumin were not able to tolerate the load of the 9 layers of material and the 3D printed flower and mountain shapes collapsed under their own weight.

Recently, many studies have shown that printed shape stability is closely related to rheological properties, especially the G′ and τ₀ of materials. The materials with higher G′ and τ₀ showed better shape retention. The τ₀ shows the minimum force necessary for material extrusion and G′ reflects the mechanical strength of mixtures. For developing structures through 3D printing, G′ and τ₀ are critical for supporting sequentially deposited layers and stability of printed shape. Liu et al. [32] determined τ₀ of 312.16 (Pa), which had good extrudability and shape retention for the extrusion-based printer. In the present study, the samples with the best 3D shapes were produced from formulas fortified with SPAH and cricket protein, having high values for τ₀ (486 Pa–535 Pa) and G′ (717 Pa–995 Pa). The mixtures with either SPAH or cricket protein had higher τ₀ and G′ and were strong enough to support the deposited layers and hold the printed structures. The samples with different concentrations of egg albumin had low τ₀ (A3 290 Pa and A5 270 Pa) and G’ (A3 96 Pa and A5 88 Pa), lower even than the control sample, and did not possess enough mechanical strength to uphold complex shapes with many layers, resulting in the deformation of the printed shape and the poor resolution supporting structure observed in formulations including egg albumin (Figure 4).

## 4. Discussion

The 3D printing process can be separated into three stages: 1—extrusion, 2—recovery, and 3—self-support. These stages are directly connected with the rheological properties, including viscosity, yield stress and elastic modulus (G′), and viscous modulus (G″) [33]. Extrusion correlates with yield stress and it is worth noting that, although the smaller the yield stress, the easier for the material to be extruded, it also implied that the mechanical strength of the material may be unfavorable for the subsequent self-support and deformation resistance of the material extrusion [34].

Adding SPAH and cricket protein improved the rheological characteristics, showed stronger mechanical properties, and furthered printability. SPAH and cricket proteins were found to be ideal ingredients for 3D food printing.

Proteins and polysaccharides interact with each other and can change the structure and properties of food formulations. These interactions are attracting a lot of attention in the food industry because food structure and stability are dependent on them [35].

Conventionally, when complexes of protein-polysaccharide are formed, their functional properties are potentially better than those of the proteins and polysaccharides alone. However, characteristics depend on several parameters such as protein and polysaccharide type and concentrations, pH, temperature, and the concentration of cations present in the solution, which can form complexes or thermodynamic incompatibilities [36,37].

Mixing two biopolymers in a solution, such as a polysaccharide and a protein, can build interactions that may present two possibilities: the interaction of the two biopolymers can be segregative (the biopolymers repel each other and are denoted as incompatible) or associative (the biopolymers attract one another) [38]. Hence, in this work, with the addition of SPAH and cricket protein, the behavior of the polysaccharide-protein mixture was associative and could create a complexation mixture. In opposition, egg albumin protein behavior with polysaccharides in the condition of this work created a segregative mixture that causes incompatibility. Conditions for incompatibility are dependent on the structure and composition of the biopolymer pair [39].

The samples with egg albumin showed low values for the rheological parameters compared to the control sample due to the segregation in the polysaccharide-protein mixture produced by their incompatibility. Grinberg and Tolstoguzov [40] demonstrated that incompatibility is strongly dependent upon the conformational state of the proteins and is enhanced by protein denaturation. Egg albumin has a very low transition temperature and can be more susceptible to suffering incompatibility effects associated with its denaturation.

## 5. Conclusions

It is a new challenge to create a nutritional formulation that is customized, extrudable, and consistent for complex shapes. In conclusion, the printability and print stability of pureed food can be improved by adding protein. This study demonstrated the applicability of food ingredients (e.g., soy protein, cricket protein, and egg albumin protein) in 3D food printing, which is a starting point for the future development of healthy, nutritional, and customized foods. The addition of soy and cricket protein (in the doses tested) significantly increased all the rheological values (yield stress, viscosity, G′, and G″). The consistency and firmness according to the extrusion force test were increased significantly in the case of using soy and cricket protein, which provided an accurate printing result and a stable geometry for multilayer complex shapes. Additionally, the rheological values and the consistency obtained from the extrusion force test increased with the amount of soy protein. Best printing results were achieved by using soy and cricket proteins which showed good fluidity with higher G′, G″, and τ₀. On the other hand, the addition of egg albumin could not support the designed structure during the 3D printing process and showed weaker rheological and textural properties because of protein incompatibility due to protein denaturation in the conditions of this study.

## Figures and Tables

**Table 1:** Puree potato’s nutritional value per 100 g of product.

Energy	1475 KJ/348 Kcal
Fat	0.8 g
Carbohydrates	75 g
Fiber	6.8 g
Proteins	7.4 g
Salt	0.06 g
Moisture	16.7 g

**Table 2:** Protein information.

Protein	Soy Protein (S)		Cricket Protein (C)		Egg Albumin (A)	
**Protein % in recipe**	3	5	3	5	3	5
**Protein/100 g product**	2.9	4.6	2.7	4.4	2.9	4.6
**Energy Kcal/100 g product**	59.3	67.6	61.3	71.5	58.9	66.8

**Table 3:** The Bingham model η = ηᵨ + τ₀/ɣ̇ parameters for the different proteins at different concentrations.

Samples	Yield Point (Pa)	ηᵨ (Pa·s)	τ₀ (Pa)
CS	620 ^bc^	105 ^bc^	302 ^b^
S3	636 ^bc^	254 ^b^	469 ^ab^
S5	981 ^a^	451 ^a^	535 ^a^
C3	742 ^bc^	302 ^ab^	486 ^ab^
C5	917 ^b^	397 ^ab^	503 ^a^
A3	530 ^c^	87 ^c^	290 ^c^
A5	558 ^c^	92 ^c^	270 ^c^

Note: CS (Control Sample), S3 (3% Soy), S5 (5% Soy), C3 (3% Cricket), C5 (5% Cricket), A3 (3% Albumin), and A5 (5% Albumin). Different superscript letters in the same column indicate a significant difference (*p* < 0.05).

**Table 4:** Storage modulus (G′), loss modulus (G″), and tan δ at 10 Hz for different proteins at different concentrations.

Samples	G′ (Pa)	G″ (Pa)	TANGENT δ (−)
CS	686 ^b^	106 ^bc^	0.16 ^ab^
S3	717 ^b^	121 ^b^	0.16 ^ab^
S5	995 ^a^	163 ^a^	0.13 ^b^
C3	816 ^ab^	130 ^ab^	0.15 ^b^
C5	827 ^ab^	131 ^ab^	0.15 ^b^
A3	661 ^bc^	96 ^c^	0.19 ^a^
A5	404 ^c^	88 ^c^	0.19 ^a^

Note: CS (Control Sample), S3 (3% Soy), S5 (5% Soy), C3 (3% Cricket), C5 (5% Cricket), A3 (3% Albumin), and A5 (5% Albumin). Different superscript letters in the same column indicate a significant difference (*p* < 0.05).

**Table 5:** Textural properties of 3D printed samples for different proteins at different concentrations.

Samples	Hardness (N)	Adhesiveness (N·s)	Cohesiveness (−)	Springiness (−)	Gumminess (N)	Chewiness (−)	Resilience (−)
CS	2.68 ^a^	−4.16 ^b^	0.005 ^a^	0.009 ^a^	2.21 ^a^	2.08 ^a^	0.0007 ^a^
S3	3.36 ^a^	−5.55 ^a^	0.006 ^a^	0.009 ^a^	2.30 ^a^	2.11 ^a^	0.0007 ^a^
S5	3.34 ^a^	−5.70 ^a^	0.006 ^a^	0.009 ^a^	2.39 ^a^	2.21 ^a^	0.0006 ^a^
C3	3.61 ^a^	−5.75 ^a^	0.006 ^a^	0.009 ^a^	2.52 ^a^	2.35 ^a^	0.0006 ^a^
C5	3.58 ^a^	−6.19 ^a^	0.006 ^a^	0.009 ^a^	2.55 ^a^	2.36 ^a^	0.0006 ^a^
A3	2.72 ^a^	−3.80 ^c^	0.005 ^a^	0.008 ^a^	2.12 ^a^	2.01 ^a^	0.0007 ^a^
A5	2.79 ^a^	−3.73 ^c^	0.005 ^a^	0.009 ^a^	2.32 ^a^	2.04 ^a^	0.0007 ^a^

Note: CS (Control Sample), S3 (3% Soy), S5 (5% Soy), C3 (3% Cricket), C5 (5% Cricket), A3 (3% Albumin), and A5 (5% Albumin). Different superscript letters in the same column indicate a significant difference (*p* < 0.05).

## Data Availability

The data presented in this study are available within the article.
